# A comparative study of esketamine-dexmedetomidine and sufentanil-dexmedetomidine for sedation and analgesia in lung tumor percutaneous radiofrequency ablation (PRFA): a randomized double-blind clinical trial

**DOI:** 10.1186/s12871-023-02266-y

**Published:** 2023-09-08

**Authors:** Zhonglan Lin, Shuxin Li, Yun Zhou, Xinlei Lu, Bin Yang, Zhengwei Yu, Yuan Cheng, Jianliang Sun

**Affiliations:** 1 The Fourth Clinical School of Medicine, Zhejiang Chinese Medical University, Hangzhou, 310006 China; 2 Department of Anesthesiology, Zhejiang Provincial Hospital of Chinese Medicine, Hangzhou, 310006 China

**Keywords:** Esketamine, Dexmedetomidine, Sedation, Analgesia, Lung tumor radiofrequency ablation

## Abstract

**Objective:**

To observe and evaluate the effectiveness and safety of Esketamine or Sufentanil combined with Dexmedetomidine for sedation and analgesia in lung tumor percutaneous radiofrequency ablation (PRFA) to provide a clinical basis for the optimization of sedation and analgesia in lung tumor PRFA protocols outside the operating room.

**Methods:**

In this trial, 44 patients aged 37 to 84 undergoing lung tumor PRFA were enrolled and assigned to Group E (n = 22, Esketamine 0.2 mg/kg) or Group S (n = 22,Sufentanil 0.1 μg/kg ). Dexmedetomidine was infused intravenously as a sedative in both groups. The modified observer’s assessment of alertness and sedation scale (MOAAS), physical movement pain scale, intraoperative vital signs, anesthesia recovery time, radiologist and patient satisfaction rates, incidence of respiratory depression, and incidence of postoperative nausea and vomiting were recorded.

**Results:**

Although there was no significant difference in the physical movement pain scale, blood oxygen saturation or incidence of perioperative adverse events between the two groups during ablation, the MOAAS, mean arterial pressure (MAP) and heart rate (HR) were higher in Group E than in Group S. The anesthesia recovery time was shorter in Group E than in Group S, and radiologist satisfaction was better in Group E than in Group S, but there was no significant difference between the two groups in terms of patient satisfaction.

**Conclusion:**

Esketamine or Sufentanil combined with Dexmedetomidine is safe for lung tumor PRFA. However, in elderly patients with multiple underlying diseases, low-dose Esketamine combined with Dexmedetomidine has fewer hemodynamic effects on patients, milder respiratory depression, shorter recovery time, and better radiologist satisfaction because of its better controllability of sedation depth.

**Trial registration:**

Chinese Clinical Trial Registry (Registration number#ChiCTR ChiCTR21000500 21); Date of Registration: 16/08/2021

## Introduction

Primary lung cancer is one of the most common malignant tumors worldwide and one of the most common causes of cancer death [1]. Surgery is regarded as the treatment of choice, but it may be risky in patients with advanced age, cardiopulmonary insufficiency, intestinal obstruction, severe hypertension, severe chronic obstructive pulmonary disease (COPD) and other serious diseases. Lung tumor PRFA is a minimally invasive approach intended to treat primary or metastatic solid tumors via thermal tissue destruction, resulting in fewer complications than traditional surgery [2, 3]. It is necessary to ensure that the patient is moderately sedated for analgesia during the perioperative period to maintain a relatively fixed position yet to cooperate with the operation, especially when the distance between the tumor and the pleura is less than a centimeter. The patient often feels severe pain due to stimulation of the pleural nerve by thermal deposition [4]. Therefore, effective analgesia during PRFA is especially important.

The traditional choice of opioids in combination with Propofol or Benzodiazepines for invasive procedures in interventional radiology suites is too great a sedative effect for patients to cooperate with the operation, such as breath-holding or lowering the respiratory rate during the puncture process, which increases the risk of bleeding, pneumothorax and air embolism [5].

A single low dose of Esketamine combined with Dexmedetomidine can maintain a mild level of sedation while meeting pain relief needs, which would be well suited for invasive procedures in interventional radiology suites. However, to our knowledge, there has been limited evaluation of Esketamine combined with Dexmedetomidine for lung tumor PRFA sedation.

This study aimed to observe and evaluate the effectiveness and safety of Esketamine or Sufentanil combined with Dexmedetomidine for sedation and analgesia in lung tumor PRFA and to compare the advantages and disadvantages of these two drug regimens.

## Materials and methods

### Study design and setting

This prospective, randomized and double-blind clinical study was conducted at Hangzhou First People’s Hospital from Mar 2022 to Feb 2023. The study was approved by the Research Ethics Committee of Hangzhou First People’s Hospital (IIT-20220217-0021) and registered in the Chinese Clinical Trial Registry (ChiCTR2100050021). Written informed consent was obtained from all subjects participating in the trial.

### Study population

In this study, we included consecutive patients who (1) were ≥ 18 years of age; whose (2) ASA classification was between I-III; whose (3) BMI index (body mass index) did not exceed 30; and who (4) were able to sign the informed consent form independently. We excluded patients who (1) were receiving continuous infusion of sedative agents and pain relievers; (2) had severe ischemic heart disease, mental illness, pregnancy, seizures, or increased intracranial pressure; and (3) had a history of allergic reactions to the drugs planned for the study.

A total of 46 patients with lung cancer were observed from Mar 2022 to Feb 2023, and 44 patients met the inclusion criteria and were included in the final analysis.

### Study protocol

This study was grouped by a random number table, and 44 patients were randomized into Group S (n = 22) or Group E (n = 22). All patients were instructed to avoid eating for 8 h and to avoid drinking for 4 h before surgery, and none of them took any preoperative medication. The surgery was performed by 2 experienced radiologists, and the sedation protocol and grouping were not known to the patients, operators or data collectors.

The patients were asked to lie down in a supine position on the examination bed after entering the CT room and were monitored continuously with ECG, pulse oximetry, respiratory rate, noninvasive blood pressure (NIBP) and BIS values. Oxygen therapy was administered at a rate of 2 L/min though a nasal catheter.

All patients were anesthetized by the same experienced anesthetist who was unaware of the groups in the study. Both groups were sedated in the same way, with a loading dose of Dexmedetomidine (1 μg/kg) micropumped within 10 min from the start of the surgery and a maintenance dose of 0.6 μg/kg·h during the surgery. After infiltration with 2% lignocaine prior to puncture, Group S received Sufentanil(a bolus of 0.1 μg/kg, iv), and Group E received Esketamine(a bolus of 0.2 mg/kg, iv). These drugs were injected three minutes prior to ablation. We aimed to maintain a mild to moderate level of sedation based on ASA criteria equivalent to the Modified Observer’s Assessment of Alertness and Sedation (MOAAS) scale of 2 to 4 (Table 1). The patient’s sedation level was assessed intraoperatively using MOAAS every 5 min. At the same time, the patient would have a physical movement pain scale of 0 to 1 (Table 2). If a patient’s MOAAS suddenly fell to 1, Dexmedetomidine was stopped, and the MOAAS was assessed again 1 min later. If the MOAAS scale was > 2 and the patient was still too responsive to tolerate the ablation, additional sedation was provided with Sufentanil 0.05 μg/kg (Group S) or Esketamine 0.1 mg/kg (Group E). All anesthetic drugs were stopped immediately when the ablation had been completed. After the surgery, patients were taken back to the ward once their Aldrete score was ≥ 9.

**Table 1:** Modified observer’s assessment of alertness and sedation scale (MOAAS)

Score	Responsiveness
5	Responds readily to name spoken in a normal tone
4	Lethargic response to name spoken in a normal tone
3	Responds only after name is called loudly and/or repeatedly
2	Responds only after mild prodding or shaking
1	Responds only after painful trapezius squeeze
0	No response after painful trapezius squeeze

**Table 2:** Physical movement pain scale

Score	Responsiveness
0	No response after painful trapezius squeeze
1	Painful expressions, informing of soreness or slight physical movements
2	Moderate body movements, which affect surgical procedures
3	Vigorous body movements prevent the operation from being continued

The MOAAS, blood pressure, heart rate, SpO_2_ and BIS values were recorded at eight time points: 5 min after entering the CT room (baseline value T0), When Dexmedetomidine was injected with a loading dose (T1), after Sufentanil or Esketamine was injected (T2), at the start of ablation (T3), 5 min after the start of ablation (T4), 10 min after the start of ablation (T5), 5 min after the end of the surgery (T6), and 10 min after the end of the surgery (T7). The physical movement pain scale was recorded from T2 to T7.

Sedation-related adverse events included oxygen desaturation, hypotension, hypertension, sinus bradycardia and postoperative nausea and vomiting. Respiratory depression was defined as SpO_2_<95%, reguiring manual airway opening. Severe hypoxia was defined as SpO_2_<75% or SpO_2_<90% for more than 60 s, the need for mask pressure oxygen to assist ventilation, and tracheal intubation if necessary [6].

Surgery-related adverse events included pneumothorax, choking and coughing, pulmonary hemorrhage, thoracodynia, absorption of heat and lung infection.

The total ablation time, duration of procedures, recovery time, length of hospital stay, and satisfaction scores of the radiologists and patients were also recorded (4 for very satisfied, 3 for fairly satisfied, 2 for fair, 1 for unsatisfied).

### Statistical methods

The statistical software SPSS version 26.0 (IBM SPSS Inc., Armonk, United States) and GraphPad Prism version 8.0 were used for data processing. The data are expressed as the mean ± standard deviation (mean ± SD), median (interquartile spacing) or frequency (percentage) where appropriate. Independent samples *t* tests were used to evaluate normally distributed continuous data. Two-way repeated-measures ANOVA was used for repeated-measures normally distributed continuous data. The Mann‒Whitney *U* test was used for nonnormally distributed data. The chi-squared or Fisher’s exact test was used for categorical data. *P* < 0.05 was accepted as a statistically significant difference.

The main index of this study was the MOAA/S between the two groups of patients 5 min after the ablation. According to the pretest results, Group E: MOAA/S mean value = 3.93, standard deviation (SD) = 0.7, Group S: MOAA/S mean value = 3.05, SD = 0.71, with bilateral α = 0.05 and β = 0.2, n = 20 was calculated by the formula $n = \frac{{{{({Z_\alpha } + {Z_\beta })}^2}(1{ + ^1}{/_k}){\sigma ^2}}}{{{\delta ^2}}},\,k = 1.$ If we calculate the percentage of lost visits at 10%, we will end up with 22 cases in each of the two groups.

## Results

### Disposition and baseline characteristics of subjects

**Fig. 1:**
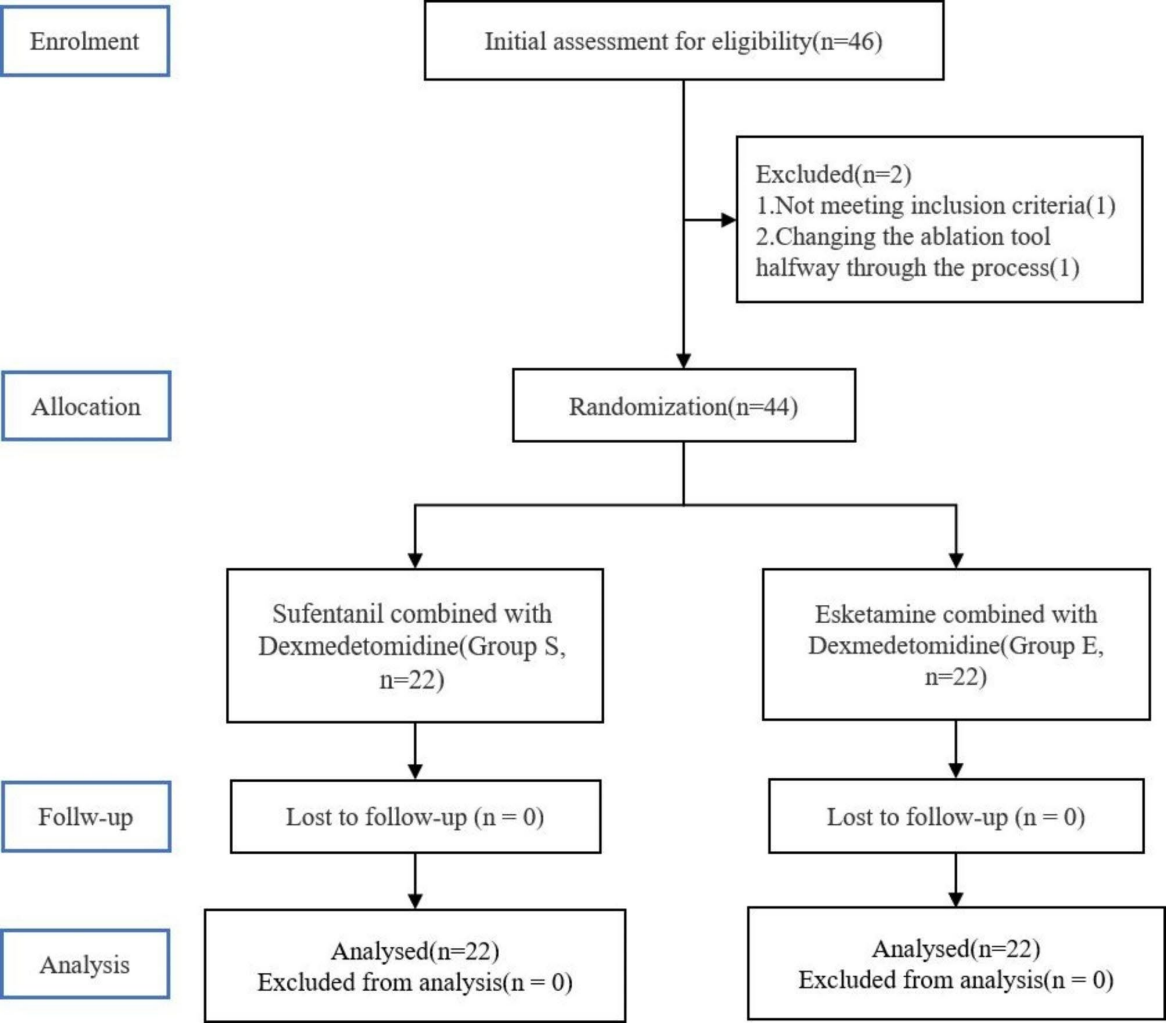
Flow diagram for the trial

From March 2022 to February 2023, a total of 46 patients aged between 37 and 80 years were enrolled in the study. Of these, 2 patients were excluded due to refusal to sign informed consent (n = 1) or a halfway change in the type of surgery (n = 1). A total of 44 patients (27 males and 17 females) were randomized to the study, 22 in Group E and 22 in Group S. Patients followed the standardized anesthetic and surgical procedures and completed all evaluation items. Finally, we performed a statistical analysis of the data from 44 patients and created a flow diagram for the trial (Fig. 1).

The demographic characteristics, ASA classification, vital signs and comorbidities were relatively comparable between the two groups. A total of 51 pulmonary lesions were ablated in this study. One case was not completely ablated because the lesion was too large and closely adjacent to the blood vessels, and the total ablation rate was 50 cases (98.4%).

There was no significant difference in the long diameter of the target lesion, distance between the lung tumors and the pleura, or ablation tumor numbers between Group S and Group E (Table 1). A total of 34 (77.3%) patients with secondary lung tumors and 10 patients with lung nodules considered to be primary lung tumors without pathological confirmation were studied, and all patients underwent PRFA following puncture biopsy of the lesions.

### Sedation and analgesia scores and hemodynamic parameters

**Fig. 2:**
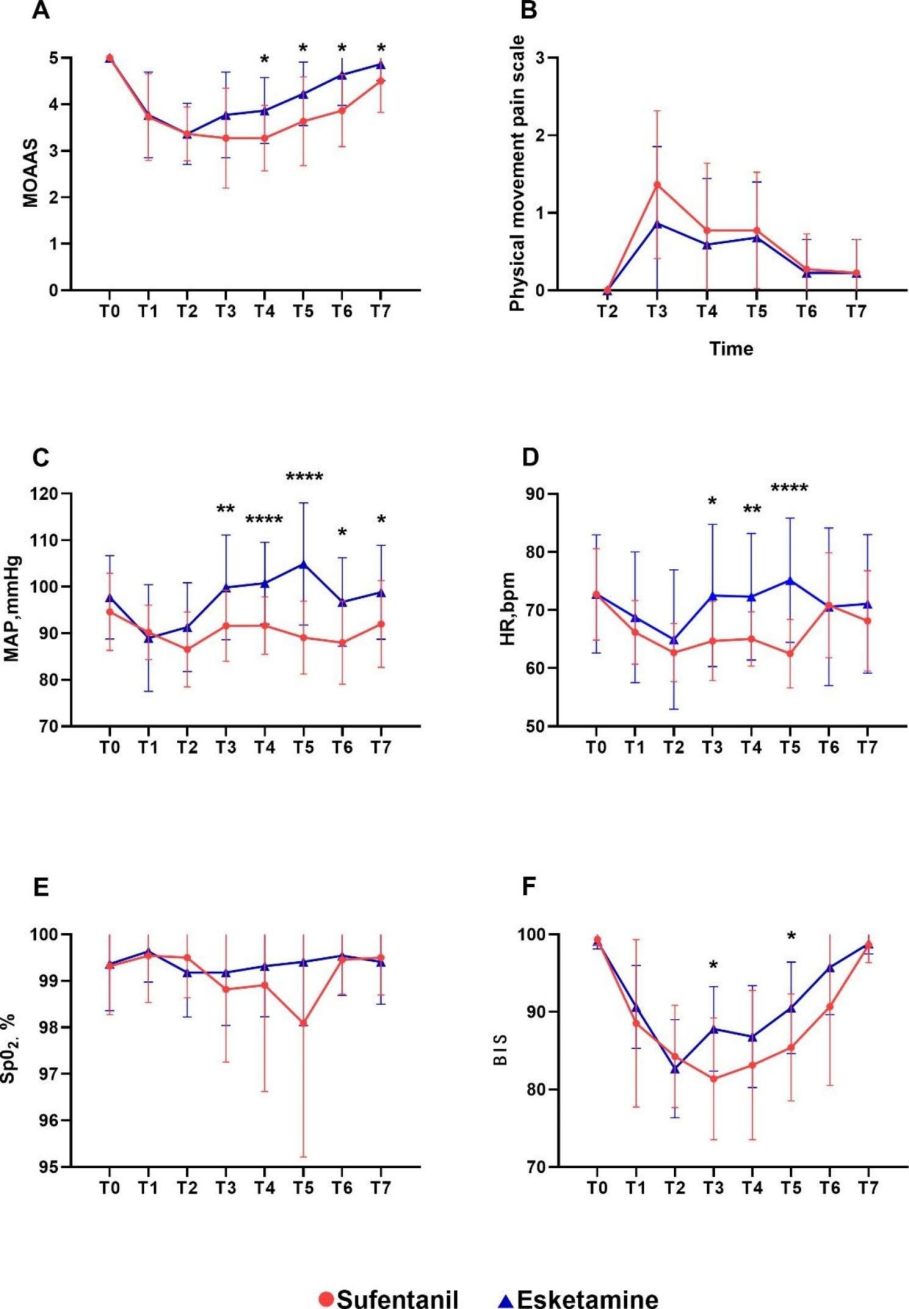
Sedation and analgesia scores and hemodynamic parameters. **A** MOAAS. **B** Physical movement pain scale. **C** MAP. **D** HR. **E** SpO_2_. **F** BIS. MOAAS, The modified observer’s assessment of alertness and sedation scale; MAP, mean arterial pressure; HR, heart rate; SpO_2_, saturation of peripheral oxygen. *P < 0.05, **P < 0.01, ***P < 0.001, ****P < 0.0001.

Two-way repeated-measures ANOVA showed that there was a statistically significant difference in MOAAS between the two groups (*P* = 0.001; Fig. 2A) and a statistically significant difference between the two groups at the T4 to T7 time points (*P* = 0.008, 0.023, 0.001, 0.030). The BIS values were also significantly different between the two groups (*P* = 0.007; Fig. 2F), and at T3 and T5, the two groups were significantly different (*P* = 0.003, 0.011). The physical movement pain scale was not significantly different between the two groups(*P* > 0.05; Fig. 2B).

Two-way repeated-measures ANOVA showed a significant difference in MAP between the two groups (*P* = 0.001; Fig. 2C) and at the T3 to T7 time points (*P* = 0.007, 0.000, 0.003, 0.025). The HR was significantly different between the two groups at the T3-T5 time points (*P* = 0.012, 0.006, 0.000,Fig. 2D). There was no significant difference in oxygen saturation between the two groups (Fig. 2E).

### Perioperative adverse events

#### Sedation-related adverse events

The total incidence of respiratory adverse events in this study was 18.2% (4/44), with 2 cases of respiratory depression and 1 case of severe hypoxemia in Group S and 1 case of respiratory depression in Group E. The total incidence of hypotension was 15.9% (7/44), including 5 cases in Group S and 2 cases in Group E. The total incidence of hypertension was 11.36% (5/44), with all 5 cases occurring in Group E. The total incidence of postoperative nausea and vomiting (PONV) was 22.73% (10/44), with 6 cases in Group S and 4 cases in Group E(Table 3).

**Table 3:** Baseline characteristics of the patients and details of PRFA

Characteristics	Sufen group(n = 22)	Esket group(n = 22)	Statistical significance
**Demographics**			
Age	67.50 ± 8.85	64.59 ± 9.62	0.361
Gender,Male(n%)	13(59.1%)	14(63.6%)	0.759
Body Mass Index(kg/m^2^)	22.47 ± 3.20	22.66 ± 3.12	0.847
**ASA status, I/II/III**	7/12/2003	10/9/2003	0.728
**Cause of tumor, Secondary tumor(n%)**	16(72.7%)	17(77.3%)	0.56
**Long diameter of target lesion (mm)**	16.18 ± 4.72	15.32 ± 5.01	0.322
**Distance from pleura**(≤ 20mm)	17(77.3%)	14(63.6%)	
**Ablation tumor numbers (n%)**			
1	19(86.4%)	18(81.8%)	1
2	3(13.6%)	4(18.2%)	1
**Comorbidities**			
Hypertension	9(40.9%)	7(31.8%)	0.531
Pulmonary Disease	7(31.8%)	5(22.7%)	0.498
Chronic Bronchitis	4(18.2%)	2(9.1%)	0.66
COPD	3(13.6%)	3(13.6%)	1
Diabetes	5(22.7%)	3(13.5%)	0.696
Smoker	7(31.8%)	5(22.7%)	0.498
Alcohol	3(13.6%)	2(9.1%)	1

Two-sample Student’s t-test was used for continuous data and Pearson’s chi-squared or Fisher’s exact test were used for categorical data. S, Sufentanil-Dexmedetomidine; E, Esketamine-Dexmedetomidine; ASA status, American Society of Anesthesiologists; COPD, Chronic obstructive pulmonary disease

#### Surgery-related adverse events

The total incidence of perioperative pneumothorax was 29.56% (13/44). Five patients were given chest drains after surgery, and their pneumothorax almost resolved within 3–7 days. The total incidence of choking and coughing was 11.36% (5/44). One patient had slight hemoptysis, which was considered to be postpuncture pulmonary hemorrhage that disappeared after a short period of hemostasis and anti-infective treatment. One patient developed postablation pulmonary absorption fever and received no specific treatment. The other 3 patients presented with pulmonary infection with fever, which resolved after anti-infective treatment for one week(Table 4).

**Table 4:** Perioperative adverse events

Perioperative adverse events	Sufentanil group(n = 22)	Esketamine group(n = 22)	Statistical significance
**Sedation-related adverse events**			
1) Respiratory depression (SpO_2_ < 95%, requiring manual airway opening)	2(9.09%)	1(4.55%)	1
2) Severe hypoxia (SpO_2_ < 75% or SpO_2_ < 90% for more than 60 seconds)	1(4.55%)	0	1
3) Hypotension (blood pressure<20% of baseline, or blood pressure<90/60mmHg)	5(22.73%)	2(9.09%)	0.41
4) Hypertension (blood pressure>20% of baseline or blood pressure > 180/100mmHg)	0	5(22.73%)	0.057
5) Bradycardia	0	0	
6) PONV	6(27.27%)	4(18.2%)	0.472
**Surgery-related adverse events**			
Pneumothorax	6(27.27%)	7(31.82%)	0.741
choking and coughing	2(9.09%)	3(13.64%)	1
Pulmonary haemorrhage	1(4.55%)	0	1
Thoracodynia	1(4.55%)	0	1
Post-operation fever			
Absorption of heat	1(4.55%)	0	1
Lung infection	2(9.09%)	1(4.55%)	1

Data are expressed as n(%).S, Sufentanil-Dexmedetomidine; E, Esketamine-Dexmedetomidine

### Outcome measures and satisfaction

There was no significant difference in total ablation time (Group S = 4.5 (3.87, 7.25) min and Group E = 4.63 (4.50, 5.63) min; *P* = 0.953) or duration of procedures (Group S = 74.23 ± 21.82 min and Group E = 74.91 ± 24.07 min; *P* = 0.922). The recovery time (Group S = 9.05 ± 2.72 min, Group E = 6.5 ± 2.20 min; *P* = 0.001) was remarkably different, and there was no awakening delay in either group. Surgeon satisfaction was significantly higher in Group E than in Group S (*P* = 0.045). Patient satisfaction was not significantly different between the two groups(Table 5).

**Table 5:** Outcome measures and satisfaction

Outcome measures	Group S (n = 22)	Group E (n = 22)	Statistical significance
Total ablation time(min)	4.5(3.87,7.25)	4.63(4.50,5.63)	0.953
Duration of procedures (min)	74.23 ± 21.82	74.91 ± 24.07	0.922
Recovery time(min)	9.05 ± 2.72	6.5 ± 2.20	0.01
Length of hospital stay(day)	9.19 ± 4.12	7.45 ± 3.56	0.144
Satisfaction scores of the radiologists	3.4 ± 0.6	3.7 ± 0.5	0.045
Satisfaction scores of the patients	3.7 ± 0.5	3.8 ± 0.4	0.163

Data are expressed as mean ± SD. S,Sufentanil-Dexmedetomidine; E,Esketamine-Dexmedetomidine

## Discussion

This study shows that a single low dose of Esketamine or Sufentanil combined with Dexmedetomidine is both safe and effective for sedation in lung tumor PRFA. Esketamine combined with dexmedetomidine was superior to sufentanil combined with dexmedetomidine because of the better anesthetic controllability, smoother hemodynamics and shorter recovery time.

PRFA is based on hyperthermia, which causes localized tumor coagulation and necrosis [7]. Therefore, intraprocedural and postprocedural pain are inevitable. In addition, for greater puncture accuracy, we expect the patient to match the shallow, slow breathing rate during the localization of the puncture needle. There is no consensus on the best method of anesthesia, and Hoffmann’s study [8] showed no significant difference between endotracheal general anesthesia and procedural sedation on the success rate, outcome and complication rate of PRFA of lung tumors. Regardless of the choice of anesthesia, our aim is to relieve or eliminate pain and discomfort (including stress, anxiety and fear) during the patient’s treatment and to prevent or reduce intraoperative body movements that may impair the treatment or lead to serious complications.

Propofol is still the most commonly used sedative drug for short procedures outside the operating room, but it has almost no analgesic effect and may suffer from hemodynamic fluctuation, insufficient arousal and respiratory depression. The majority of these surgical procedures are for elderly and palliative care patients with malignancy, who are more likely to have cardiac and cardiopulmonary diseases. We should be cautious about the type and dosage of sedative drugs we use.

Dexmedetomidine is regularly used for pre-operative and/or intra-operative and other procedural sedation in non-intubated patients, and for sedation of patients who are on ventilators in the intensive care unit. In previous studies on the dosage of Dexmedetomidine for awake fiberoptic nasotracheal intubation(AFOI), the loading dose (0.4–1.5 μg/kg over 10 or 15 min) and the maintenance dose (0-0.7 μg/kg·h) provided an acceptable level of sedation for patients to remain responsive and cooperative [9]. In this study, to unnecessarily avoid oversedation and hemodynamic instability, the loading dose of Dexmedetomidine was 1 μg/kg for 10 min, and the maintenance dose was 0.6 μg/kg·h. Dexmedetomidine is a highly selective α_2_-adrenergic agonist with sedative and anxiolytic effects that is noninhibitory to respiration, inhibits sympathetic excitability and reduces sympathetic tension. Recent studies have found that Dexmedetomidine activates Dopamine Neurons in the Ventral Tegmental Area Attenuates the Depth of Sedation [10]. Given its limited analgesic effect, Dexmedetomidine is not suitable as a single drug for invasive procedures, and it may be a good option in combination with another analgesic drug.

Sufentanil is a kind of μ-opioid receptor agonist that is approximately 5 to 10-fold more potent in analgesia than Fentanyl [11]. Sufentanil as an induction and maintenance drug for general endotracheal anesthesia(GETA) in the operating room, but concerns about respiratory depression limit its widespread use for procedural sedation. Low doses (0.1 to 2 μg/kg) of Sufentanil are commonly used for general anesthesia, induction and minimally invasive surgery in outpatient settings [12]. The dose of Sufentanil used in this study was 0.1 μg/kg. Esketamine is the dextroisomer of ketamine, which has a higher affinity for N-methyl-D-aspartate (NMDA) receptors and a stronger sedative and analgesic effect [13]. Furthermore, some studies have shown that subanesthetic doses (0.2-0.3 mg/Kg) of Esketamine already have analgesic effects [14]. The dose of Esketamine used in this study was 0.2 mg/kg.

In clinical practice, synergistic or additive effects are often observed when sedative-hypnotic drugs are used in combination with opioid analgesics [15]. This trial showed that the analgesic effect was similar in Group S and Group E, but the level of sedation was significantly lighter in Group E than in Group S. Studies by Scheinin et al. [16] and Horvath et al. [17] have shown that Dexmedetomidine enhances the analgesic effect of opioids and Ketamine, but their sedative effects are unclear. Weerink’s study showed that Remifentanil seems to have no effect on Dexmedetomidine -induced sedation [15]. Tose’s study showed that Ketamine enhances the activity of norepinephrine (NE) in the locus coeruleus (LC) through its effect on orexins (OXs). NE is essential for maintaining wakefulness, which immediately increases arousal and reduces nonrapid eye movement sleep (NREMS) upon Ketamine anesthesia [18, 19]. It was hypothesized that Esketamine would not deepen sedation levels while providing analgesia, which is consistent with the findings of this study. We observed higher BIS values in Group E than in Group S during ablation, especially at the T3 and T5 time points, which is consistent with the outcomes of the MOAAS. This seems to corroborate the lighter level of sedation in Group E than in Group S. It has also been suggested that Ketamine can increase BIS values and thus affect the judging of anesthetic depth [20].

In addition to widely used in general anaesthesia surgery, acute pain and chronic pain treatments, Esketamine has good clinical applications in the treatment of mental illness, emergency and critical illnesses.The effectiveness of Dexmedetomidine in combination with Ketamine for procedural sedation is well documented [21]. Dexmedetomidine limits Ketamine-induced tachycardia, hypertension and salivation; Ketamine alleviates the adverse cardiovascular system effects of Dexmedetomidine, sympathetic depression and bradycardia, thereby enhancing the hemodynamic stability of patients [22]. In this study, mean arterial and heart rates in Group E during ablation were closer to preprocedure levels, which benefited elderly patients with a lower heart rate or a more fragile cardiovascular system.

Although the depth of sedation is relatively mild, multiple complications have the potential to occur in the perioperative period. Respiratory events due to poor oxygen supply or ventilation are the most common adverse events during extraoperative anesthesia [23]. Activation of μ-opioid receptors in the central nervous system is associated with adverse effects such as oversedation, respiratory depression and PONV [24]. Esketamine stimulates breathing by increasing carbon dioxide-sensitive ventilation [23]. Studies by Drummond et al. on lingual muscle activity and airway obstruction during sedation with Midazolam and Ketamine showed that Ketamine had a beneficial effect on airway patency [25]. There were more patients with respiratory depression in Group S than in Group E. There were three patients in Group S and one patient in Group E. The comparison between the two groups was not statistically significant. This is not consistent with the findings of previous studies, and we believe this may be due to the small sample size.

It has been shown that the inflammatory response to necrotic tissue after ablation is a risk factor for PONV. In addition, preoperative nausea and vomiting, female sex, a history of PONV or motion sickness, age (> 50 years old), chemotherapy, and nonsmokers are all risk factors for PONV [26]. According to Apfel’s risk stratification model, the incidence of PONV increases to more than 60% for patients with three or more risk factors [27]. In this study, the incidence of PONV in Group S and Group E was 27.3% and 18.2%, respectively, with no significant difference between the two groups. Opioids are a key trigger for PONV. A rise in the plasma concentrations of catecholamines is known to induce PONV, and Esketamine has a central sympathomimetic effect and hence may increase the incidence of PONV [28].

There are some notable limitations to our study. First, we used only a single dose of Esketamine or Sufentanil, which may not be the most appropriate dose. Second, as a single center and a small number of randomized controlled trials (RCTs), further randomized controlled trials in multiple centers and more patients are needed to confirm our findings. Third, the large interference in monitoring the respiratory rate prevents us from accurately monitoring the patient’s respiratory rate. Finally, as a clinical study, further studies are needed to confirm the drug interaction between Esketamine and Dexmedetomidine.

## Conclusions

In summary, a single low dose of Esketamine or Sufentanil combined with Dexmedetomidine are both safe and feasible methods that can successfully complete lung tumor PRFA. However, we prefer a low dose of Esketamine combined with Dexmedetomidine in elderly people with more basic disease, which has less hemodynamic impact, less respiratory depression and shorter recovery time from the anesthesia state. Radiologists prefer Esketamine in combination with dexmedetomidine because of its better controllability of sedation.

## Data Availability

The datasets generated and analyzed during the current study are available from the corresponding author upon reasonable request.
